# Small changes of femoral torsion in varus or valgus distal femoral osteotomy using patient-specific instruments

**DOI:** 10.1007/s00264-025-06415-5

**Published:** 2025-01-29

**Authors:** Lukas Jud, Georgios Neopoulos, Sandro Hodel, Lazaros Vlachopoulos, Sandro F. Fucentese

**Affiliations:** Universitätsklinik Balgrist, Zurich, Switzerland

**Keywords:** Knee osteoarthritis, Hinge fracture, Knee osteotomy, Distal femoral osteotomy, DFO

## Abstract

**Purpose:**

Hinge fractures show a relatively high incidence in varus and valgus distal femoral osteotomy (DFO) and can lead to delayed- or non-union. Another observed complication of a hinge fracture is an unintentional change of the postoperative femoral torsion of up to + 9.5° in conventionally performed DFO. We hypothesize that the change of femoral torsion in case of a hinge fracture is less pronounced when DFO is performed using patient-specific instruments (PSI) compared to the literature of conventionally performed DFO.

**Methods:**

All patients who underwent varus or valgus DFO using PSI from January 2014 to September 2023 were included. Radiographs and computed tomography (CT) scans were used to screen for hinge fractures. Pre- and postoperative femoral torsion was measured in CT.

**Results:**

Thirty-five medial closing-wedge DFO (MCW-DFO), 27 lateral closing-wedge DFO (LCW-DFO), and 27 lateral opening-wedge DFO (LOW-DFO) were included, resulting in a total of 89 included osteotomies. A total of 55 hinge fractures (61.8%) were observed. The femoral torsion changed significantly from 20.5° ± 7.7° to 15.5° ± 8.1° (*p* < 0.001) in LOW-DFO with a hinge fracture, whereas the other two techniques showed no significant change of femoral torsion.

**Conclusion:**

The use of PSI in varus and valgus DFO showed only small changes of the postoperative femoral torsion, even in case of a hinge fracture. The change of femoral torsion was depending on the type of DFO and was only significant in LOW-DFO, however, not exceeding a mean change of 5°.

## Introduction

Varus and valgus distal femoral osteotomy (DFO) is frequently used in younger patients with unicompartimental knee osteoarthritis or patients with patellofemoral instability to correct the underlying deformity [1–4]. Commonly used surgical techniques for correcting the coronal malalignment include lateral opening- and closing-wedge, or medial closing-wedge DFO [2, 5, 6]. While opening-wedge osteotomy is considered technically easier to perform and includes the advantage of height restoration, it bears an increased risk of delayed union compared to the technically more demanding closing-wedge technique, which allows a faster rehabilitation and healing of the osteotomy [7–9]. A dreaded complication of both techniques, opening- and closing-wedge DFO, is a fracture of the cortical hinge, with reported incidences of up to 46% and 70%, respectively [10–12]. Hinge fractures increase the risk for delayed- and non-union due to increased torsional load on the bone-implant construct [13, 14]. Furthermore, hinge fractures are recognized to cause unintended rotational changes of the femur, with an increase of the femoral torsion of + 9.5° on average [15]. Such an increase in femoral torsion may negatively affect the patellofemoral joint, as increased femoral torsion is known to contribute to patellofemoral degeneration [16, 17], and instability [18, 19]. However, a basic requisite in varus or valgus DFO is an appropriate planning and a precise surgical execution. One proposed approach to optimize the surgical execution is the use of patient-specific instruments (PSI) [20–23]. This study aimed to investigate the incidence of hinge fracture in varus and valgus DFO using PSI and to evaluate the influence of hinge fractures on postoperative femoral torsion using this technique. We hypothesized that the use of the PSI, with its additional stabilization given by the PSI reduction guide, would result in low postoperative deviation of femoral torsion in case of a hinge fracture.

## Material and methods

The local ethical committee approved this study (Zurich Cantonal Ethics Commission, BASEC-Nr. 2023–00389) and all patients gave their informed consent.

All patients who underwent varus or valgus DFO using PSI from January 2014 to September 2023 at our institution were included. According to our standard protocol, patients receive conventional antero-posterior and lateral knee radiographs, long leg radiographs (LLR) and a computed tomography (CT) scan preoperatively and at 4.5 months postoperatively. At six weeks postoperatively, conventional antero-posterior and lateral knee radiographs are obtained. Patients without available full radiological dataset were excluded.

### Surgical technique

**Fig. 1:**
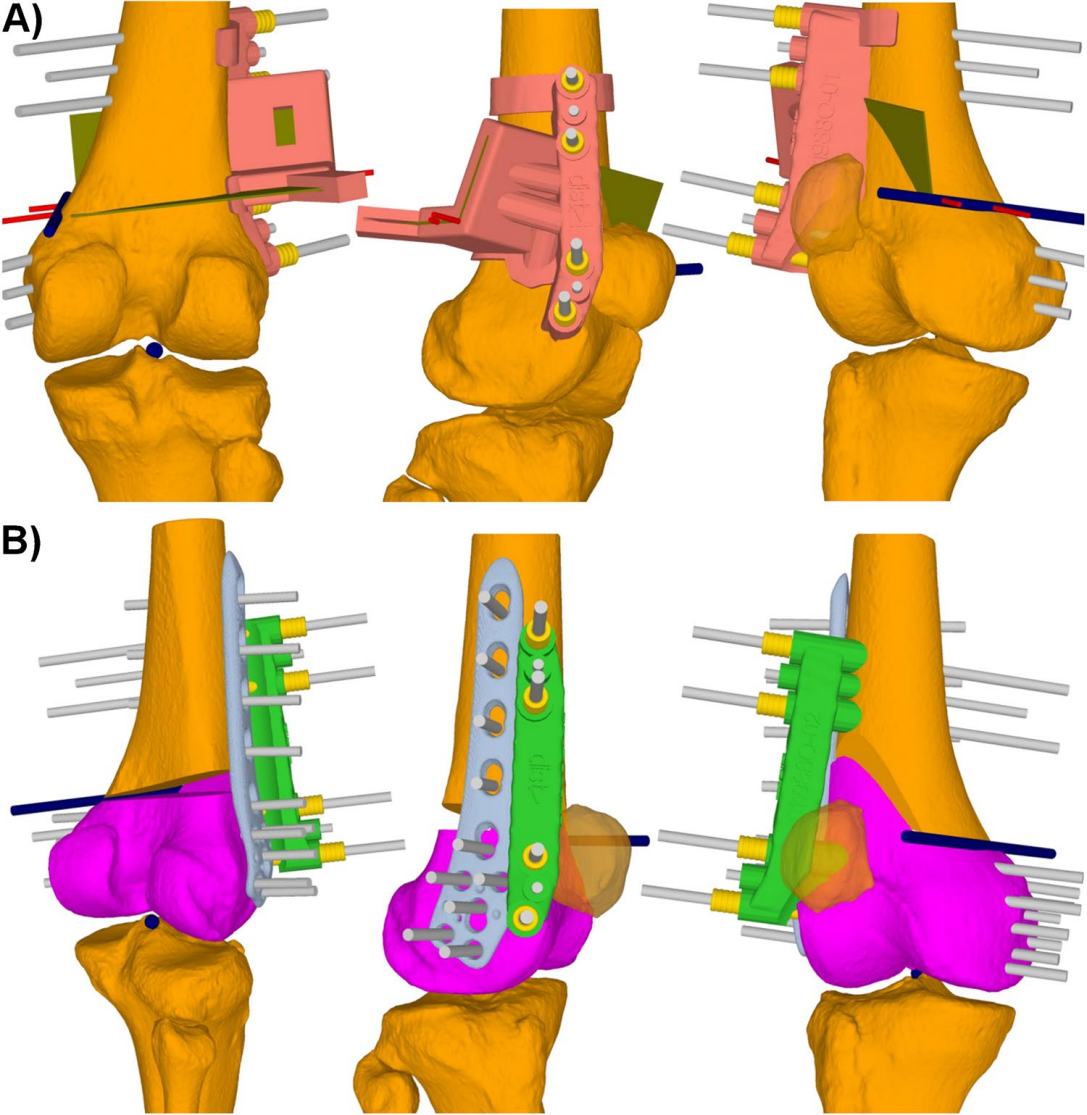
Osteotomy guide (light red) in **A**. Preliminary fixation is performed by two k-wires (red). After confirmation of the correct guide position by fluoroscopy, the definitive fixation is performed using four Schanz pins (grey). Afterwards, the orientation of the biplanar osteotomy and the cutting depth is defined by the osetotomy guide. After performing the osteotomy, the osteotomy guide can be removed and the reduction guide (green) can be placed over the four Schanz pins which is visualized in **B**. The placement of the reduction guide results in the desired opening of the osteotomy. Finally, plate fixation of the osteotomy can be performed. The dark blue axis represents the hinge axis

PSI were used for all performed surgeries. Preoperative planning of the PSI was similar to other PSI osteotomies around the knee [20, 22, 24]. A lateral or medial subvastus approach was used to expose the distal femur. After identification of bony landmarks, the osteotomy guide was positioned and preliminary fixated using k-wires (Fig. 1). Confirmation of the correct guide position was performed using fluoroscopy and definitive fixation of the guide was performed using four Schanz pins. Afterwards, the biplanar osteotomy was performed. The osteotomy guide defined the accurate osteotomy orientation and the required cutting depth. After completing the osteotomy, the osteotomy guide was removed, leaving the four Schanz pins in place. The reduction guide was then placed over the four Schanz pins, resulting in the aimed opening or closing of the osteotomy (Fig. 1). Finally, fixation of the osteotomy was performed using a lateral or medial distal femur plate (TomoFix, DePuy Synthes, Oberdorf, Switzerland) and fluoroscopy was used to document the implant position.

### Radiological assessment

**Fig. 2:**
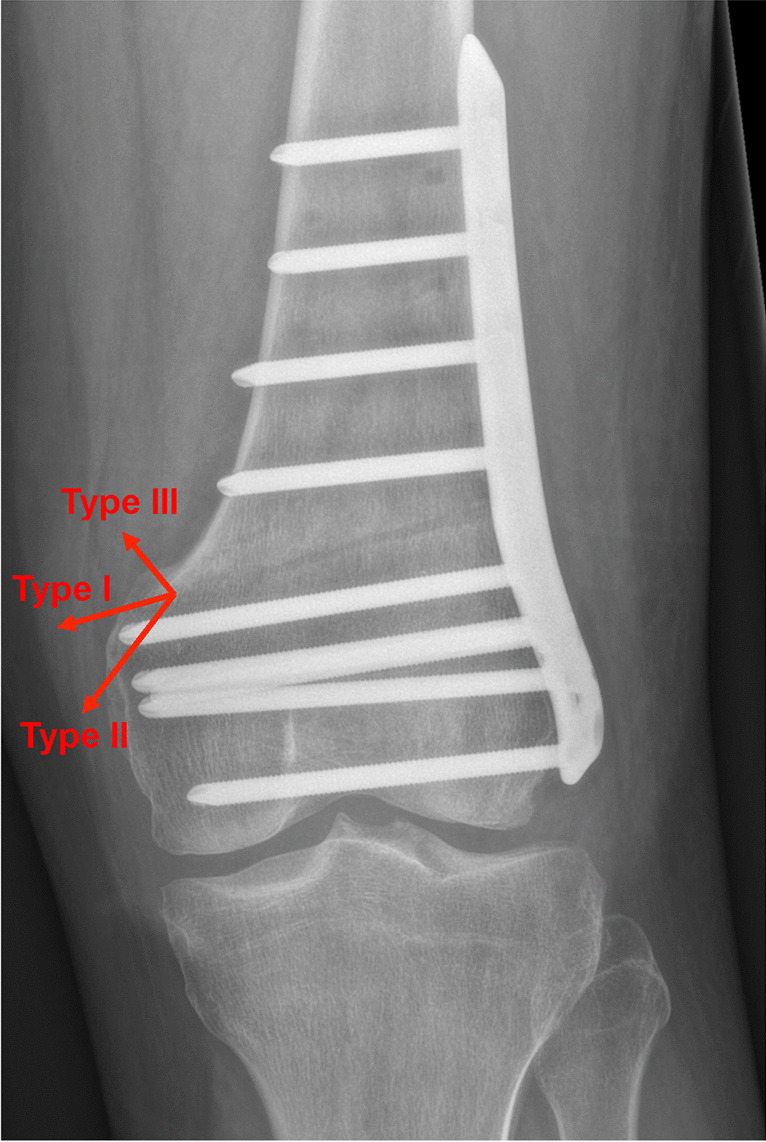
Hinge fracture classification. Hinge fractures were classified in extension (Type I), distal (Type II), and proximal (Type III) fractures

Femoral torsion was measured in pre- and postoperative CT scan according to the method described by Waidelich [25]. Femoral antetorsion was handled as positive values. The hip-knee-ankle angle (HKA) was measured using LLR preoperatively and at 4.5 months postoperatively [26]. Intraoperative fluoroscopic images, conventional knee radiographs at six weeks, and LLR and CT scan at 4.5 months postoperatively were screened for hinge fractures. Hinge fractures were classified in extension (Type I), distal (Type II), and proximal (Type III) fractures (Fig. 2) [27].

### Statistical analysis

Continuous variables were reported as mean and standard deviation (SD). Mean change of femoral torsion was calculated for each case as postoperative femoral torsion—preoperative femoral torsion. Normality of distribution was tested using the Shapiro–Wilk test. Accordingly, the paired t-test or the Wilcoxon-test was applied to assess differences between pre- and postoperative measurements within groups and the unpaired t-test or the Mann–Whitney-U-test was applied to assess differences between different groups. All statistical analyses were performed in SPSS for Windows (Version 29.0, SPSS Inc., Chicago, Illinois). Statistical significance was set at *p* < 0.05.

## Results

The patient demographic characteristics are summarized in Table 1. An overview of pre- and postoperative HKA measurements, and planned and achieved HKA corrections is given in Table 2.

**Table 1:** Patient demographic characteristics

Demographic characteristics	
n	89 knees, 82 patients
Sex	40 female, 42 male
Side	51 right knee, 38 left knee
Age	37.0 ± 13.1 years
BMI	28.0 ± 5.1 kg/m^2^

**Table 2:** Overview of pre- and postoperative hip-knee-ankle angle (HKA) measurements, and planned and achieved HKA corrections

	All (*n* = 89)	No hinge fracture (*n* = 34)		Hinge fracture (*n* = 55)	
Preoperative HKA (°)	1.1 ± 8.0 (−19.8 to 15.8)	−1.2 ± 8.0 (−16.0 to 9.3)		2.5 ± 7.6 (−19.8 to 15.8)	
Postoperative HKA (°)	0.2 ± 2.0 (−8.6 to 5.3)	0.3 ± 2.0 (−4.7 to 5.3)		−0.5 ± 3.0 (−8.6 to 4.8)	
Planned HKA correction (°)	−1.3 ± 8.3 (−10.0 to 16.3)	−1.1 ± 9.1 (−9.1 to 15.9)	*p* = n.s	−2.9 ± 7.4 (−10.0 to 16.3)	*p* = n.s
Reached HKA correction (°)	−1.3 ± 8.5 (−13.3 to 16.9)	−1.5 ± 8.7 (−9.3 to 16.9)		−3.1 ± 8.0 (−13.3 to 16.9)	
Absolute difference planned versus reached HKA correction (°)	1.9 ± 1.5 (0.0 to 6.3)	1.6 ± 1.1 (0.0 to 3.7)		2.1 ± 1.7 (0.0 to 6.3)	
		*p* = n.s			

The study cohort included 35 medial closing-wedge DFO (MCW-DFO), 27 lateral closing-wedge DFO (LCW-DFO), and 27 lateral opening-wedge DFO (LOW-DFO) with a total of 89 osteotomies. Overall, a hinge fracture was observed in 55 knees (61.8%), which included 25 hinge fractures in MCW-DFO (71.4%), 13 hinge fractures in LCW-DFO (48.1%), and 17 hinge fractures in LOW-DFO (63%). In 35 knees the hinge fracture was already visible in the intraoperative fluoroscopy, whereas in 16 knees it was first visible in the conventional knee radiographs  six weeks postoperatively, and in four knees in the CT scan at 4.5 months postoperatively. Thirty-nine hinge fractures corresponded to a Type I (20 MCW-DFO, 9 LCW-DFO, 10 LOW-DFO) and 16 to a Type III (5 MCW-DFO, 4 LCW-DFO, 7 LOW-DFO) hinge fracture. No Type II hinge fracture was observed.

In regards of the three different DFO techniques, a significant difference between pre- and postoperative femoral torsion was only seen for LOW-DFO (*p* < 0.001). An overview of pre- and postoperative femoral torsion measurements is given in Table 3.

**Table 3:** Overview of pre- and postoperative femoral torsion measurements

	Femoral-torsion preop (°)	Femoral-torsion postop (°)	*p*-value
All (*n* = 89)	15.2 ± 9.1 (−13.2 to 30.2)	13.7 ± 9.2 (−10.3 to 29.9)	< 0.001
No hinge fracture (*n* = 34)	14.9 ± 8.7 (−6.2 to 29.3)	14.2 ± 8.9 (−3.7 to 29.3)	n.s
Hinge fracture (*n* = 55)	15.4 ± 9.3 (−13.2 to 30.2)	13.5 ± 9.4 (−10.3 to 29.9)	0.002
Type I hinge fracture (*n* = 39)	14.9 ± 10.3 (−13.2 to 30.2)	12.8 ± 9.7 (−10.3 to 29.9)	0.007
Type III hinge fracture (*n* = 16)	16.8 ± 6.5 (3.5 to 26.5)	15.1 ± 8.6 (−5.5 to 25.5)	n.s
MCW-DFO with hinge fracture (*n* = 25)	11.7 ± 10.2 (−13.2 to 28.8)	9.9 ± 10.1 (−10.3 to 29.9)	n.s
LCW-DFO with hinge fracture (*n* = 13)	16.0 ± 6.1 (8.6 to 26.7)	17.6 ± 7.3 (5.5 to 25.5)	n.s
LOW-DFO with hinge fracture (*n* = 17)	20.5 ± 7.7 (3.5 to 30.2)	15.5 ± 8.1 (−5.5 to 24.0)	< 0.001

*preop*: preoperative; postop: postoperative; *DFO*: distal femoral osteotomy; *MCW*: medial closing-wedge; *LCW*: lateral closing-wedge; *LOW*: lateral opening-wedge

Regarding mean postoperative changes of femoral torsion, a significant difference was seen between all included knees with a hinge fracture and knees without a hinge fracture (*p* = 0.033), between LOW-DFO and MCW-DFO (*p* = 0.048), and between LOW-DFO and LCW-DFO (*p* < 0.001). An overview of postoperative mean changes of femoral torsion is given in Table 4.

**Table 4:** Overview of mean postoperative changes of femoral torsion

	Mean change of femoral-torsion postop (°)		
All (*n* = 89)	−1.5 ± 4.4 (−11.1 to 12.9)		
No hinge fracture (*n* = 34)	−0.7 ± 2.3 (−4.0 to 4.4)	*p* = 0.033	
Hinge fracture (*n* = 55)	−2.0 ± 5.3 (−11.1 to 12.9)		
Type I hinge fracture (*n* = 39)	−2.1 ± 5.5 (−11.1 to 12.9)	*p* = n.s	
Type III hinge fracture (*n* = 16)	−1.7 ± 4.9 (−9.0 to 11.0)		
MCW-DFO with hinge fracture (*n* = 25)	−1.7 ± 5.5 (−11.1 to 12.4)	*p* = n.s	*p* = 0.048
LCW-DFO with hinge fracture (*n* = 13)	1.6 ± 5.3 (−4.5 to 12.9)	*p* < 0.001	
LOW-DFO with hinge fracture (*n* = 17)	−4.9 ± 3.0 (−10.9 to 0.8)		

*postop*: postoperative; *DFO*: distal femoral osteotomy; *MCW*: medial closing-wedge; *LCW*: lateral closing-wedge; *LOW*: lateral opening-wedge

## Discussion

The most important finding of this study is that in varus and valgus DFO using the PSI technique, only small changes of the postoperative femoral torsion were observed, regardless of a concomitant hinge fracture. The degree of the postoperative change of femoral torsion was different in reference of the used DFO technique. It was only significant in case of LOW-DFO with a mean change of −4.9° (*p* < 0.001). Therefore, the hypothesis of this study could be confirmed.

Hinge fractures are frequently observed in varus and valgus DFO with reported incidences of up to 70% [12]. Different attempts have been made to avoid this complication, such as the use of a protective hinge-wire or optimization of the position of the hinge, but hinge fractures still occur [27, 28]. The main reason why this complication is such a dreaded situation is the reduced bone-implant construct stiffness in case of a hinge fracture [13, 14, 29]. Ultimately, the reduced stability of the osteotomy can lead to a delayed consolidation with increased rates of mal- and nonunion [12, 30–32]. Another observed complication of hinge fractures in valgus DFO is a change of the postoperative femoral torsion. Nakayama et al. demonstrated an increase of + 9.5° of femoral torsion in 36 knees with a conventionally performed biplanar LCW-DFO, compared to an increase of + 1.6° in knees without a hinge fracture [15]. It can be assumed that such an increase of nearly 10° of femoral torsion may negatively affect the patellofemoral joint in terms of degeneration or instability [16–19]. In our cohort, knees with a hinge fracture in LCW-DFO showed no significant change of femoral torsion in case of a hinge fracture, but a slight increase of the postoperative femoral torsion of + 1.6° was also observed in these cases, which is clearly lower than described by Nakayama et al. Considering the three different varus and valgus DFO techniques included in our study, LCW-DFO showed the least postoperative change of femoral torsion, respectively demonstrated the most stable postoperative femoral torsion. The only technique with a significant change of the femoral torsion in case of a hinge fracture from the preoperative to the postoperative situation was the LOW-DFO (*p* < 0.001). A mean decrease of femoral torsion of −4.9° was observed in hinge fractures using this technique. Concluding to the results of our study with the least change of postoperative femoral torsion in LCW-DFO, it has to be assumed that using the conventional technique for LOW-DFO, a hinge fracture may even results in larger changes of postoperative femoral torsion than the reported + 9.5° in conventionally performed LCW-DFO. One possibility to reduce postoperative changes of femoral torsion may be the use of a biplanar osteotomy, in which the anterior flange of the osteotomy may withstand a rotational movement of the distal fragment. However, biplanar osteotomies have also been used in the study of Nakayama et al. [15]. Using our PSI technique, a biplanar osteotomy was also performed. The osteotomy was further stabilized with a reduction guide (Fig. 1), which likely maintains the correct position even in the case of a hinge fracture. Besides the here presented advantage of reduces changes of the postoperative femoral torsion in case of a hinge fracture, the use of PSI have also been proven to increase the accuracy of the mechanical axis correction in LOW-DFO compared to the conventional technique [33].

Another finding of this study that should be discussed is the overall high incidence of hinge fractures of 61.8% using our PSI technique. The incidence in the MCW-DFO group was even higher with 71.4%, whereas the lowest incidence was observed in the LCW-DFO group with 48.1%. However, the incidence of hinge fractures in our collective per technique is comparable to others in the literature [10–12]. Furthermore it has to be mentioned that CT was used to detect hinge fractures in our study, and different previous studies, reporting lower hinge fracture rates, used only conventional radiographs for detecting hinge fractures [3, 30].

This study should be interpreted in lights of its potential limitations. Only patients who underwent varus and valgus DFO using the PSI technique have been included and the observed changes of postoperative femoral torsion were only compared to values of the conventional technique from literature. It would have been favorable to have a control group that underwent varus and valgus DFO using the conventional technique. The reason is the lack of a conventional control group with available postoperative CT scan at our institution. Another limitation is that different surgeons performed the included procedures in this study. However, both senior surgeons (SF, LV) performed the majority of the surgeries. If a different surgeon performed the osteotomy, at least one of them supervised the procedure.

## Conclusion

The use of PSI in varus and valgus DFO showed only small changes of the postoperative femoral torsion, even in case of a hinge fracture. The change of femoral torsion was depending on the type of DFO and was only significant in LOW-DFO, however, not exceeding a mean change of 5°.

## Data Availability

No datasets were generated or analysed during the current study.
